# Survey of visiting hours in critical care units in English trauma centres

**DOI:** 10.1186/cc14656

**Published:** 2015-03-16

**Authors:** E Taylor, N Bunker

**Affiliations:** 1 The Royal London Hospital, London, UK

## Introduction

The purpose of this study was to assess the visiting restrictions placed on families visiting adult patients on critical care units within trauma hospitals in England. Whilst it is well recognised that high-quality care for patients is of paramount importance, we should also be aware that supporting patients' families offers long-term benefits for patient, family and hospital. In our own unit we are reviewing whether we could adopt a more flexible attitude to visiting times and assessing how to provide a more welcoming environment to relatives. To inform our own review and in order to develop a best practise approach, we surveyed all of the major trauma centres in England.

## Methods

A telephone survey on visiting times was conducted in 53 adult critical care units in trauma centres in England. A list of trauma centres was obtained from the NHS England website. All critical care units (other than obstetric high dependency units and coronary care units, unless part of a cardiothoracic critical care unit) within each hospital were surveyed. Each respondent was asked about the visiting hours, whether children were allowed to visit and how many visitors to a bed space.

## Results

Fifty-three units with between four and 75 beds and covering the whole of England were surveyed: there was a 100% response rate. Visiting hours varied between hospitals and between units within the same hospital. Nine units (17%) had open visiting hours, although most gave advice on times to avoid such as nursing handover. The majority of units (44.83%) operated restricted visiting with a median (range) of 6 (2 to 9) hours. All units allowed a maximum of two visitors to the bedspace. Children were allowed in nine units without restriction, the remaining units advised that it may not be appropriate for children to visit and it was at the discretion of the parents and medical staff.

## Conclusion

The majority of adult critical care units in England, including our own, have restricted visiting policies. Visiting policies are a source of debate amongst staff in intensive care with concerns about open visiting including increased workload and interruptions to normal routine [1]. This is consistent with the views of staff at our own unit who, in appreciative enquiry, have expressed mixed opinions about extending visiting times. Extending visiting times is only part of a wider project to improve the way relatives experience intensive care whilst ensuring both medical and nursing staff feel supported, creating an environment for optimal communication.
